# ProAct: quantifying the differential activity of biological processes in tissues, cells, and user-defined contexts

**DOI:** 10.1093/nar/gkad421

**Published:** 2023-05-19

**Authors:** Moran Sharon, Gil Gruber, Chanan M Argov, Miri Volozhinsky, Esti Yeger-Lotem

**Affiliations:** Department of Clinical Biochemistry and Pharmacology, Faculty of Health Sciences, Ben-Gurion University of the Negev, POB 653 Beer-Sheva 8410501, Israel; Department of Clinical Biochemistry and Pharmacology, Faculty of Health Sciences, Ben-Gurion University of the Negev, POB 653 Beer-Sheva 8410501, Israel; Department of Clinical Biochemistry and Pharmacology, Faculty of Health Sciences, Ben-Gurion University of the Negev, POB 653 Beer-Sheva 8410501, Israel; Department of Clinical Biochemistry and Pharmacology, Faculty of Health Sciences, Ben-Gurion University of the Negev, POB 653 Beer-Sheva 8410501, Israel; Department of Clinical Biochemistry and Pharmacology, Faculty of Health Sciences, Ben-Gurion University of the Negev, POB 653 Beer-Sheva 8410501, Israel; The National Institute for Biotechnology in the Negev, Ben-Gurion University of the Negev, POB 653 Beer-Sheva 8410501, Israel

## Abstract

The distinct functions and phenotypes of human tissues and cells derive from the activity of biological processes that varies in a context-dependent manner. Here, we present the Process Activity (ProAct) webserver that estimates the preferential activity of biological processes in tissues, cells, and other contexts. Users can upload a differential gene expression matrix measured across contexts or cells, or use a built-in matrix of differential gene expression in 34 human tissues. Per context, ProAct associates gene ontology (GO) biological processes with estimated preferential activity scores, which are inferred from the input matrix. ProAct visualizes these scores across processes, contexts, and process-associated genes. ProAct also offers potential cell-type annotations for cell subsets, by inferring them from the preferential activity of 2001 cell-type-specific processes. Thus, ProAct output can highlight the distinct functions of tissues and cell types in various contexts, and can enhance cell-type annotation efforts. The ProAct webserver is available at https://netbio.bgu.ac.il/ProAct/.

## Graphical Abstract

**Graphical Abstract:**
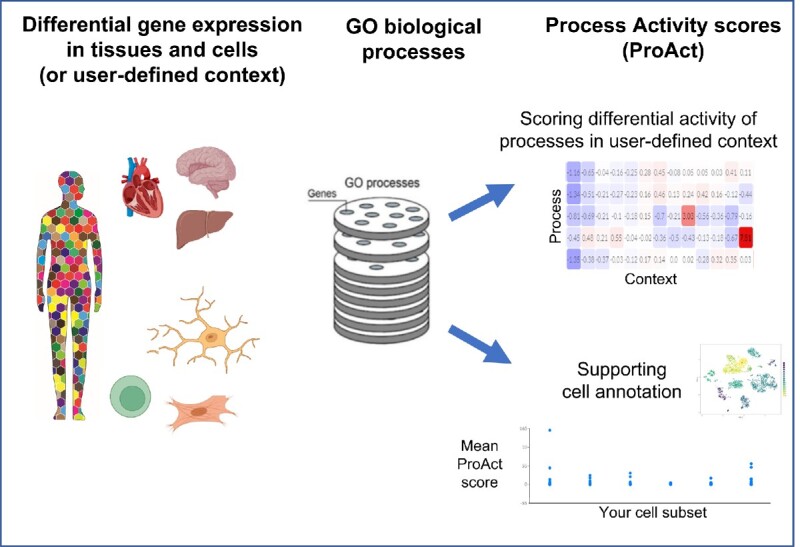

## INTRODUCTION

Huge advancements in the understanding of human phenotypes in health and disease were achieved owing to transcriptomic analyses of human tissues and cells (1,2). However, transcriptomic measurements encompass tens of thousands of distinct molecules, resulting in detailed, comprehensive datasets that are not easy to interpret. This led to the development of module- or systems-based approaches that uncover the innerworkings of physiological systems (3,4). A powerful concept that has been used widely is that of a biological process, defined as an ensemble of gene products that executes a specific biological function, such as replication or protein degradation. By connecting subsets of genes with specific functions, biological processes offer a clearer view of the state and functionality of tissues and cells (3,5).

Acknowledging their importance, biological processes have been defined and annotated by various organizations. Prominent resources include the Gene Ontology (GO) consortium (6), Reactome (7), Kyoto Encyclopedia of Genes and Genomes (8), WikiPathways (9) and Human MSigDB collections (10). Each resource contains hundreds to thousands of biological processes and their associated genes. However, information on the activity of processes in different biological contexts is not part of these resources.

A typical approach to estimate the activity or relevance of biological processes in different contexts is enrichment analysis of biological processes in transcriptomic profiles (11). It was shown that certain biological processes are active in a few biological contexts, e.g. stress response pathways in challenging environments (12,13) and differentiation processes in development (14). Other methods to estimate the activity of biological processes harnessed knowledge of the molecular interactions between process-associated genes (15,16), or focused on comparing between specific contexts, such as a tumor sample to a reference set of samples (17). Furthermore, several webtools provide process-related information and allow exploration and visualization of processes in tissues and cells (Table 1). The Reactome database (7) and the Signaling Pathways Project (SPP) (18) include representation of the expression levels of process-associated genes in tissues, however, this representation is not comparative between tissues. The HumanBase database (19) is highly informative, however its queries are limited to genes. The scTPA webserver (20) provides pathway activity profiles inferred from gene set enrichment analyses for user-defined single cell transcriptomics data, yet has limited query options and web functions.

**Table 1.** Available webtools for estimating biological process activities

Tool name	Tool type (database/ webserver)	Query type	Computational method	User input	Data type	Tissue (#); Cell types (#)	Score
Reactome (7)	database (pathway browser)	process	visual representation of expression levels	✗	transcriptomics	✓ (32); ✗	✗
HumanBase (19)	database	gene set in a tissue/cell	functional module detection	✓ gene names	transcriptomics	✓ (144 tissues and cells)	process enrichment in a functional module (q-val)
Signaling Pathways Project (18)	database	process in a tissue	gene ranking by differential expression	✗	transcriptomics, chip-seq	✓ (10); ✗	✗
scTPA (20)	webserver	process, cell subset	gene set enrichments, clustering	✓ gene expression matrix	transcriptomics	✗; ✗	enrichment-based pathway activity (Gene Set Variation Analysis)
ProAct	webserver	process(es), gene(s), tissue, cell subset	mean differential expression per process and context	✓ differential gene expression matrix	differential gene expression	✓ (34); ✓ (35)	preferential process activity

We recently reported a method to identify biological processes that were preferentially active or under-expressed in specific contexts, denoted Tissue Process Activity (TiPA) (21). TiPA was applied to GO biological processes, owing to the large number of annotated processes and genes in GO and its uniform nomenclature across organisms. Per context, TiPA associated a process with the mean differential expression of its genes relative to other contexts. We showed via analysis of 1579 processes in 34 human tissues that TiPA was able to identify tissue-specific processes, and was better than another method (17). Application of TiPA to transcriptomic profiles of 108 cell subsets (22) revealed that preferentially active processes often matched cell type identities, suggesting that they could facilitate the annotation of uncharacterized cell subsets.

**Figure 1.**
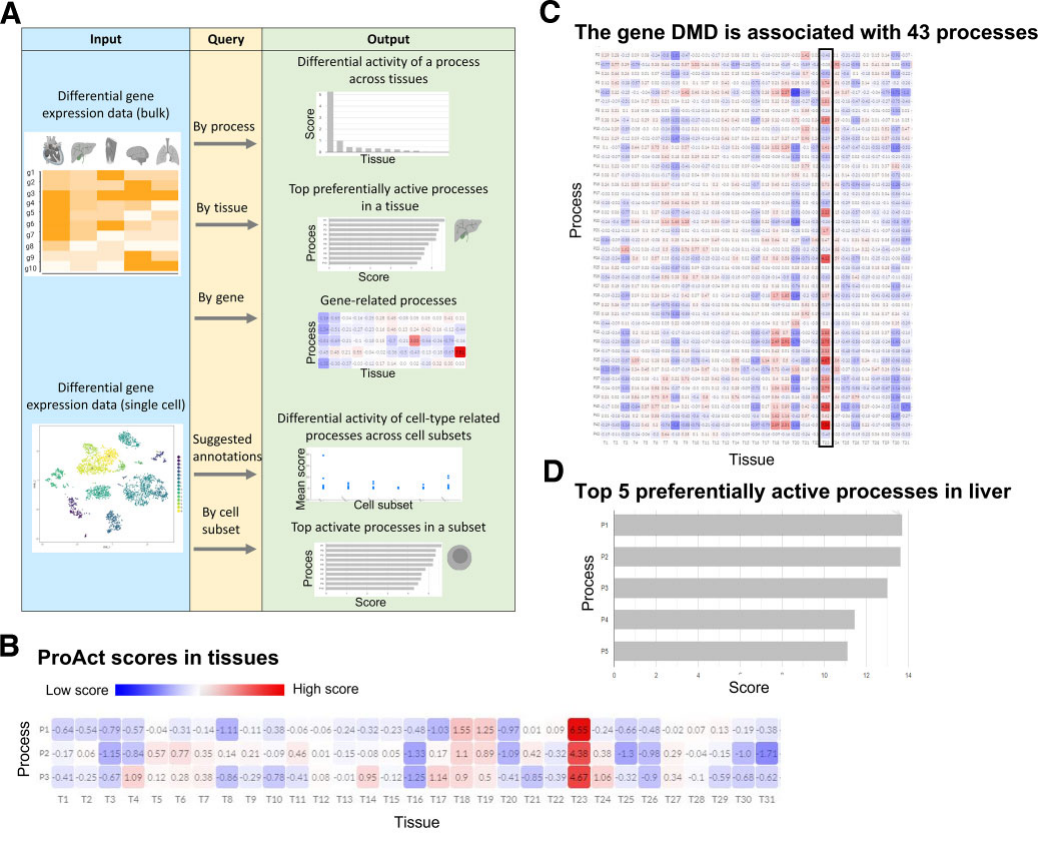
An overview of ProAct webserver functionality. (**A**) Analysis starts with a user-defined differential expression matrix across contexts, such as tissues or cell types. ProAct then computes the preferential activity of processes per context. Outputs are visualized graphically. (**B**) The preferential activity across tissues of the processes ‘skeletal muscle contraction’ (P1), ‘regulation of skeletal muscle contraction’ (P2), and ‘regulation of skeletal muscle contraction by regulation of release of sequestered calcium ion’ (P3). The highest activity of all processes was obtained in skeletal muscle (red entries, T23). (**C**) Preferentially activity of 43 processes involving the gene DMD in 34 human tissues. The black frame indicates skeletal muscle. (**D**) The five topmost preferentially active processes in liver were related to liver functions: ‘triglyceride-rich lipoprotein particle remodeling’ (P1), ‘negative regulation of very-low-density lipoprotein particle remodeling’ (P2), ‘monocarboxylic acid metabolic process’ (P3), ‘monoterpenoid metabolic process’ (P4) and ‘glyoxylate catabolic process’ (P5).

Here we report the ProAct webserver for applying the TiPA method to user-defined transcriptomics data. Given a differential expression matrix inferred from bulk or single cell transcriptomics, the webserver computes the preferential activity of GO biological processes per context. ProAct can be queried by process, context, or gene through a user-friendly interface. Its visualizations of the output allow users to quickly grasp the preferential activity of biological processes in various contexts (Figure 1). ProAct also provides precomputed preferential activities of GO biological processes and MSigDB hallmark gene sets in 34 human tissues, based on transcriptomic data from GTEx (1). Lastly, given a user-defined differential expression matrix of cell subsets, ProAct combines the preferential activity of 2001 cell-type-specific processes to offer likely cell-type annotations. ProAct is freely available at https://netbio.bgu.ac.il/ProAct/.

## MATERIALS AND METHODS

### Biological processes

Data of GO biological process terms and process-associated human genes were downloaded from Ensembl BioMart on 3 October 2020 (GRCh38.p13) (23). Data of MSigDB hallmark gene sets were downloaded from the ‘Human MSigDB Collections’ on 24 April 2023 (10).

### ProAct scores

ProAct scores (also denoted TiPA scores) were computed according to the TiPA method (21). The computation relies on (i) an input matrix of the differential expression (typically log2 fold-change values) of genes (rows) per contexts (column) and (ii) a list of processes and their associated genes. For each process *p* and context *c*, the ProAct score is set to the mean differential expression in *c* of the genes associated with *p*. There might be cases where a single or few process-associated genes are dramatically differentially expressed relative to all other process-associated genes. To reduce the impact of such outliers on a ProAct score we trimmed the mean, such that process-associated genes with the 10% most extreme differential expression values were excluded from the computation.

### ProAct scores in human tissues

ProAct webserver offers precomputed ProAct scores that estimate the preferential activity of GO processes and MSigDB hallmark gene sets in 34 human tissues. Transcriptomic profiles were obtained from GTEx (1). Brain sub-regions were grouped into six anatomically-related tissues denoted brain0-brain5 (Supplementary Table S2) (24). The GO-based precomputed dataset included ProAct scores for 6939 GO processes with 3–100 expressed genes and a z-score-derived *P*-value (21). *P*-values were adjusted for multiple hypotheses testing using Benjamini–Hochberg procedure.

### ProAct analysis of cell subsets

scRNA-sequencing profiles of fetal human tissues were obtained from (22), and included the number of reads per gene in 172 cell subsets. The differential expression matrix contained the preferential expression of each gene in each cell subset relative to all other cell subsets (25). ProAct scores were computed as described above.

### ProAct subset annotation

**Figure 2.**
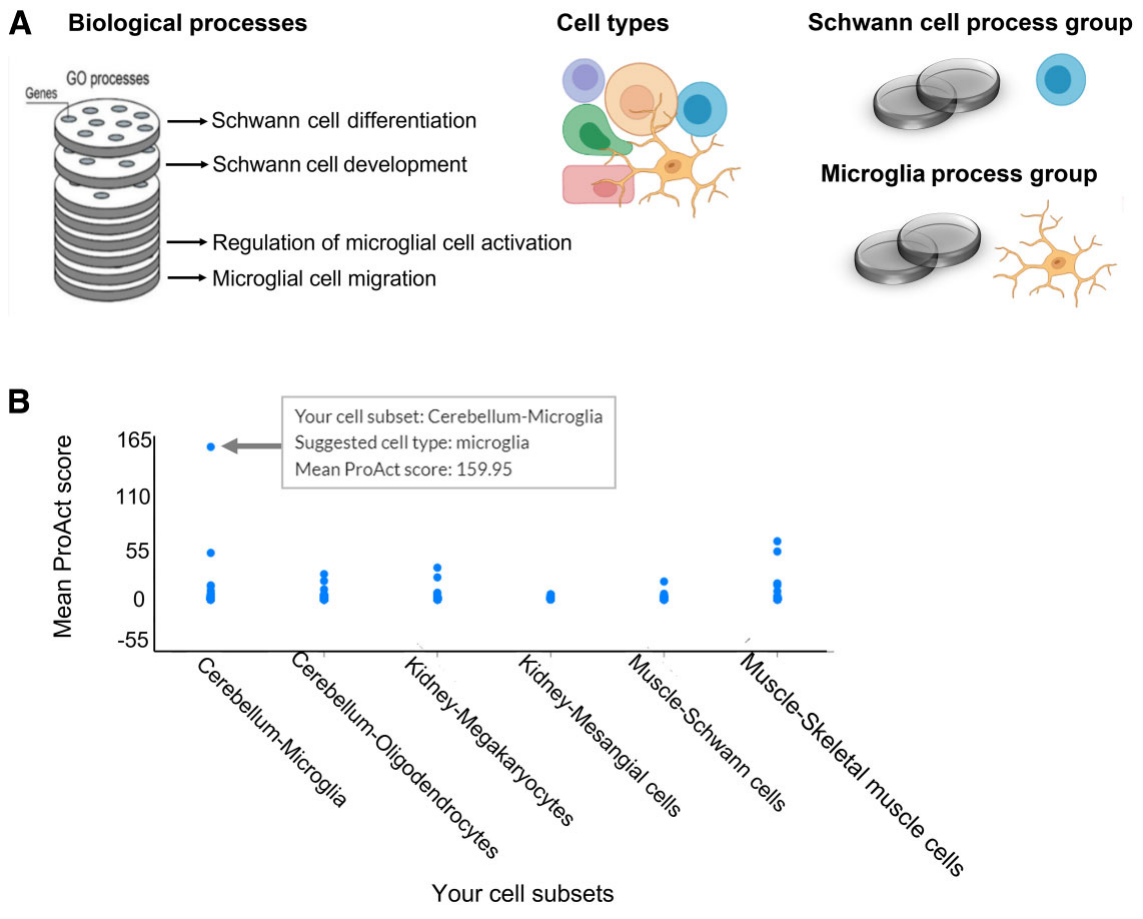
ProAct annotation of cell subsets. (**A**) GO process terms were associated with cell types and grouped accordingly into cell-type process groups. For example, the terms ‘Schwann cell differentiation’ and ‘Schwann cell development’ were associated with Schwann cells and grouped into a ‘Schwann cell process group’. The terms ‘regulation of microglial cell activation’ and ‘microglial cell migration’ were associated with microglia cells and grouped into a ‘microglia process group’. (**B**) ProAct suggested annotations for six cell types (X axis). Each dot represents the mean ProAct score (Y axis) of a distinct celltype process group. The top-ranking cell-type process groups in each cell type were indicative of cell-type identities: microglia ranked first in cerebellum-microglia cells; oligodendrocytes ranked first in cerebellum-oligodendrocytes; megakaryocytes ranked first in kidney-megakaryocytes; mesangial stem cells ranked second in kidney-mesangial cells; Schwann cells ranked first in muscle-Schwann cells; skeletal muscle cells ranked first in muscle-skeletal muscle cells.

To facilitate annotation of cell subsets, we identified cell-type-specific GO biological process terms, as described in (21). Specifically, cell types were matched with processes whose name or description contained cell-type-related words using text-mining. For example, B cells were matched with the process ‘marginal zone B cell differentiation’. Altogether, we matched between 2001 GO biological processes and 35 cell types (Supplementary Table S1, also downloadable from ProAct website). Next, GO terms that were associated with the same cell type were added to a cell-type process group (Figure 2A). For example, ‘regulation of fat cell differentiation’ and ‘fat pad development’ were added to a ‘fat cells process group’. Given an input matrix of the differential expression of genes in cell subsets, ProAct scores are computed per cell subset as described above. Next, per cell subset, the score of every cell-type process group is set to the mean ProAct scores of the processes composing the group.

## RESULTS

The ProAct webserver estimates the preferential activity of biological processes in different contexts. This is achieved by scoring a process in a given context by the mean differential expression of its genes in that context relative to other contexts (21). Hence, in a given context, positive ProAct scores indicate processes that are preferentially active, whereas negative ProAct scores indicate downregulated processes. Below we describe ProAct and its application to bulk and single cell differential expression data.

### ProAct can illuminate context-dependent processes

ProAct analysis starts with a user-defined differential expression matrix, whereby each entry corresponds to the differential expression of a gene in one context relative to other contexts. Next, per context, ProAct calculates the preferential activity of GO biological processes (Methods). ProAct can be queried by process, gene, or context (Figure 1A). The output of process queries is a heatmap representation of the preferential activity of the process across contexts (Figure 1B). The output of gene queries is the preferential activity of gene-related processes per context, and also presented by a heatmap (Figure 1C). The output of context queries includes the top preferentially active processes in that context, and is presented as a bar plot (Figure 1D). The different outputs are interactive, and users can run subsequent queries. For example, users can query ProAct by a gene, and then run a subsequent query on one of the gene-related processes.

To demonstrate the different queries, we used a dataset of differential expression of genes in 34 human tissues (21). We first queried this dataset for processes related to skeletal muscle contraction to obtain their ProAct scores across tissues. As expected, top ProAct scores were obtained in skeletal muscle, in which contraction is a key function (Figure 1B). Next, we queried the dataset by the gene Duchenne muscular dystrophy gene (DMD). The output included ProAct scores of 43 DMD-related processes in each of the 34 tissues (Figure 1C). The top preferentially active process was ‘muscle filament sliding’ in skeletal muscle, which was shown to be perturbed in the disease (32). Lastly, we queried this dataset by tissue, specifically liver. The five topmost preferentially active processes were all related to liver function, among which were ‘triglyceride-rich lipoprotein particle remodeling’, and ‘negative regulation of very-low-density lipoprotein particle remodeling’ (Figure 1D). To facilitate similar analyses, ProAct has a dedicated tissue-analysis interface and built-in differential gene expression matrix (21).

### ProAct can aid in cell-type annotation

ProAct analysis starts with a user-defined differential expression matrix of genes in different cell subsets, in a format that matches the output of the Seurat toolkit (26). Next, per cell subset, ProAct calculates the preferential activity of GO biological processes (Methods). ProAct can be queried by cell subset to reveal the top preferentially-active processes in that subset. Additionally, ProAct can suggest cell-type annotations (21). For this, we associated 2001 GO process terms with 35 matching cell types (Methods). For example, the terms ‘Schwann cell differentiation’ and ‘Schwann cell development’ were associated with Schwann cells; the term ‘Microglial cell migration’ was associated with microglia (Figure 2A). Per cell subset, ProAct associates each cell-type process group with the mean ProAct score of its processes, considered as the cell-type score. These cell-type scores are reported and presented graphically per cell subset (Figure 2B).

To demonstrate these queries, we applied ProAct to a transcriptomic dataset of six cell types from (22) (Methods). We first applied ProAct to reveal the top preferentially-active processes in megakaryocytes. Among the top five processes were ‘megakaryocyte differentiation’, ‘platelet formation’, and ‘megakaryocyte development’, all closely related to megakaryocytes functions. Next, we applied ProAct to suggest cell-type annotations (Figure 2B). In all six cell types, the correct cell-type process group ranked either second (one cell type) or first (five cell types). Hence, the top-ranking cell-type process groups of a given cell subset could illuminate its identity.

## DISCUSSION

The ProAct webserver estimates the activity of biological processes in user-defined contexts. In contrast to other tools, such as Reactome (7) or GSEA (11), ProAct does not estimate the absolute activity of a process in a given context. Instead, it estimates the activity of a process in a given context relative to all other contexts. By that, it downplays constitutively active ‘housekeeping’ processes, and highlights processes with context-dependent activity, whether preferential or downregulated. This relative, context-specific view of process activities facilitates the understanding of context-specific functions and phenotypes (21,24).

Context-specific functions are especially important for deciphering cell identities. Single cell transcriptomics revealed that the human body is composed of hundreds of cell types (2), which further divide into functionally distinct cell states (27,28) and subtypes (29). However, the identity of many of the cell subsets identified via single cell transcriptomics is unclear. Their annotation and functional characterization, which used to rely on a limited set of known marker genes, currently involves more elaborate cell signatures (30). Process-based characterization has proved useful (20,21) (Figure 2B), yet a cell subset can show preferential activity of several unrelated processes. ProAct is unique in addressing this complexity by further estimating the activity of 35 cell-type-specific process groups, resulting in more robust estimation of candidate cell types (Figure 2). ProAct could be extended to include additional and more expansive cell-type-specific process groups by using more sophisticated techniques (e.g. (31)). For example, the process ‘synaptic transmission’ is neuron-related, but this process was not associated with any cell type (Methods). Likewise, pneumocytes were associated with two processes, whereas T cells were associated with 161 processes (Supplementary Table S1). Despite limitations, when tested on 108 cell types, ProAct-based ranking of cell-type process groups filtered out irrelevant cell types and nicely captured cell type identities (21).

In summary, the ProAct webserver provides insightful and user-friendly visualizations of preferential process activities in various contexts, and can act as a supportive tool for functional characterization and annotation of newly-identified cell types. Its application to additional gene sets would offer users a broader range of biological contexts to explore.

## DATA AVAILABILITY

ProAct is freely available at https://netbio.bgu.ac.il/ProAct/. The data underlying this article are available in the article and in its online supplementary material.

## Supplementary Material

gkad421_Supplemental_File
